# The use of direct oral anticoagulants for thromboprophylaxis or treatment of cancer-associated venous thromboembolism: a meta-analysis and review of the guidelines

**DOI:** 10.1186/s12959-021-00326-2

**Published:** 2021-10-30

**Authors:** Norah S. Alsubaie, Shahad M. Al Rammah, Reema A. Alshouimi, Mohammed Y. Alzahrani, Majed S. Al Yami, Abdulaali R. Almutairi, Osamah M. Alfayez, Ghazwa B. Korayem, Omar A. Almohammed

**Affiliations:** 1 Department of Pharmacy Practice, College of Pharmacy, King Saud bin Abdulaziz University for Health Sciences, Riyadh, Saudi Arabia; 2 Saudi Food and Drug Authority, Riyadh, Saudi Arabia; 3 Department of Pharmacy Practice, College of Pharmacy, Qassim University, Qassim, Buraydah, Saudi Arabia; 4 Department of Pharmacy Practice, College of Pharmacy, Princess Nourah bint Abdulrahman University, Riyadh, Saudi Arabia; 5 Department of Clinical Pharmacy, College of Pharmacy, King Saud University, P.O. Box 2457, Riyadh, 11451 Saudi Arabia

**Keywords:** Direct oral anticoagulants, Venous thromboembolism, Thromboprophylaxis, Treatment, Cancer

## Abstract

**Background:**

Venous thromboembolism (VTE) is a common complication among patients with cancer and is one of the most common causes of increased morbidity and mortality. The use of direct oral anticoagulants (DOACs) for thromboprophylaxis and treatment of cancer-associated venous thromboembolism (CA-VTE) has been evaluated in several randomized clinical trials (RCTs). The aim of this meta-analysis was to assess efficacy and safety of using DOACs for thromboprophylaxis and treatment of CA-VTE and provide a summary for available guidelines’ recommendations.

**Methods:**

MEDLINE was searched to identify studies evaluating the use of DOACs for thromboprophylaxis or treatment in patients with cancer. Search was limited to peer-reviewed studies published in English. Studies were excluded if they were not RCTs or subgroup analyses of data derived from RCTs, if they did not report efficacy and safety data on patients with active cancer, or if they were published as an abstract. New VTE or VTE recurrence, and major or clinically relevant non-major bleeding (CRNMB) were used to assess the efficacy and safety, respectively. The Mantel-Haenszel random-effects model risk ratios (RRs) and the corresponding 95% confidence intervals (CIs) were calculated to estimate the pooled treatment effects of DOACs.

**Results:**

Four studies evaluating DOACs use for thromboprophylaxis and four – for treatment of CA-VTE were included. Thromboprophylaxis with DOACs was associated with a significant reduction in the risk of symptomatic VTE (RR = 0.58; 95%CI 0.37,0.91) but with an incremental risk of major bleeding or CRNMB (RR = 1.57; 95%CI 1.10,2.26). CA-VTE treatment with DOACs was linked with a significant reduction in VTE recurrence (RR = 0.62; 95%CI 0.44,0.87) but with an incremental risk of CRNMB (RR = 1.58; 95%CI 1.11,2.24).

**Conclusions:**

The DOACs are associated with a lower risk of symptomatic VTE and VTE recurrence, but the risk of bleeding remains a considerable concern. Clinical decisions should be made by assessing individual patient’s risk of VTE and bleeding.

**Supplementary Information:**

The online version contains supplementary material available at 10.1186/s12959-021-00326-2.

## Introduction

Venous thromboembolism (VTE), including deep vein thrombosis (DVT) and pulmonary embolism (PE), is a common complication among patients with cancer and is one of the most common causes of increased morbidity and mortality [1]. The risk factors for VTE are multifactorial and include cancer itself, chemotherapeutic agents, and patient-specific characteristics [2], such as female sex, older age, and comorbidities like diabetes and atherosclerosis [3].

The management of VTE in patients with cancer is challenging due to an increased risk of bleeding and VTE recurrence [4]. Low-molecular-weight heparin (LMWH) has been the gold standard treatment for cancer-associated VTE (CA-VTE) and is recommended over vitamin K antagonist (VKA) on the basis of evidence from several randomized controlled trials (RCTs) [5, 6]. However, considering the patient preference, availability of oral anticoagulants, cost, and the risk of thrombocytopenia, the use of this agent might not be the best option in cancer patients with VTE [7, 8].

Direct oral anticoagulants (DOACs), including apixaban, edoxaban, rivaroxaban, betrixaban, and dabigatran, have been approved for use in medical patients for VTE prophylaxis or treatment [9–11]. Since the original RCTs evaluating DOACs for VTE management included a limited number of cancer patients, separate studies were designed to investigate the use of DOACs specifically for CA-VTE. Since then, multiple RCTs have been conducted among cancer patients to evaluate the safety and efficacy of DOACs for thromboprophylaxis and treatment of CA-VTE [7, 12–17].

The PHACS trial, which assessed the use of dalteparin for the prevention of CA-VTE, provided inconclusive results because patients were noncompliant with treatment (once-daily subcutaneous injections) [18]. On the other hand, the AVERT trial concluded that apixaban significantly reduced the incidence of VTE in cancer patients, as opposed to the CASSINI trial, which showed that rivaroxaban did not lower the incidence of VTE events during the study period [12, 13]. Later on, The International Initiative on Thrombosis and Cancer (ITAC) and the American Society of Hematology (ASH) updated their guidelines to recommend the use of rivaroxaban or apixaban for thromboprophylaxis in ambulatory patients with cancer [19], and the American Society of Clinical Oncology (ASCO) guidelines stated that apixaban, rivaroxaban, or LMWH may be offered to cancer patients for thromboprophylaxis [20]. Regarding the management of CA-VTE, apixaban, edoxaban, and rivaroxaban were found to be noninferior to LMWH [15–17]. Recently, the ASCO and ITAC guidelines have added rivaroxaban and edoxaban as the first-line agents next to LMWH for the treatment of CA-VTE [20, 21]. Furthermore, the 2021 ASH guideline recommended apixaban, rivaroxaban or LMWH for initial treatment of CA-VTE [19]. The latest American College of Chest Physicians (ACCP) guidelines also recommend DOACs over LMWH for the initiation and treatment of CA-VTE [22].

The aim of this systematic review and meta-analysis was to assess the efficacy and safety of using DOACs for thromboprophylaxis and treatment of CA-VTE. We also summarized the recommendations from guidelines regarding the use of DOACs in patients with cancer for the management of CA-VTE.

## Methods

### Data sources and study selection

A systematic review was conducted using MEDLINE (from January 1st 2009 through July, 31st 2020) to identify studies evaluating the use of DOACs for thromboprophylaxis or treatment in patients with cancer and reporting VTE and bleeding events. The following search terms were used: cancer, venous thromboembolism, pulmonary embolism, low-molecular-weight heparin, enoxaparin, dalteparin, tinzaparin, factor Xa inhibitors, apixaban, betrixaban, dabigatran, edoxaban, and rivaroxaban. For CA-VTE treatment, RCTs were included, and for thromboprophylaxis, RCTs and studies with subgroup analyses reporting efficacy and safety data on patients with active cancer as well as post-hoc analyses of RCTs were included. The search was limited to peer-reviewed studies published in English. Studies were excluded if they were not RCTs or subgroup analyses of data derived from RCTs, if they did not report efficacy and safety data on patients with active cancer, or if they were published as an abstract. Each study was screened for eligibility independently by two authors.

### Outcomes

The efficacy outcomes in the meta-analysis were VTE recurrence in treatment studies (within up to 6 months of follow-up from the start of treatment) or a new VTE event in thromboprophylaxis studies (within up to 6 months of follow-up from the start of thromboprophylaxis). The safety outcomes were the incidence of major bleeding, clinically relevant nonmajor bleeding (CRNMB), and major bleeding or CRNMB events during the follow-up. Definitions of the efficacy and safety outcomes from the original trials were included in Supplementary Table S1.

### Data extraction

Data were extracted and assessed for accuracy independently by two authors (NSA and SMA) and verified by the third author (MYA). Data were extracted on a predefined data extraction form. For each study, the following data were extracted: VTE recurrence (for treatment studies) or VTE events (for thromboprophylaxis studies), symptomatic VTE, major bleeding, CRNMB, and major bleeding or CRNMB events.

### Statistical analysis

The Mantel-Haenszel random-effects model risk ratios (RRs) and the corresponding 95% confidence intervals (CIs) were calculated using the metan routine in Stata software, version 14.2 (StataCorp LLC, College Station, Texas, United States) to estimate the pooled treatment effects of DOACs. Heterogeneity was assessed using the I^2^ statistics, and the values of < 40, 30–60%, 50–90% and 75–100% were defined as low, moderate, substantial, and considerable heterogeneity, respectively [23]. The risk-of-bias assessment was conducted for each study using the Cochrane Collaboration’s tool, and a funnel plot was used to assess publication bias.

## Results

### Characteristics of the included studies

**Fig. 1:**
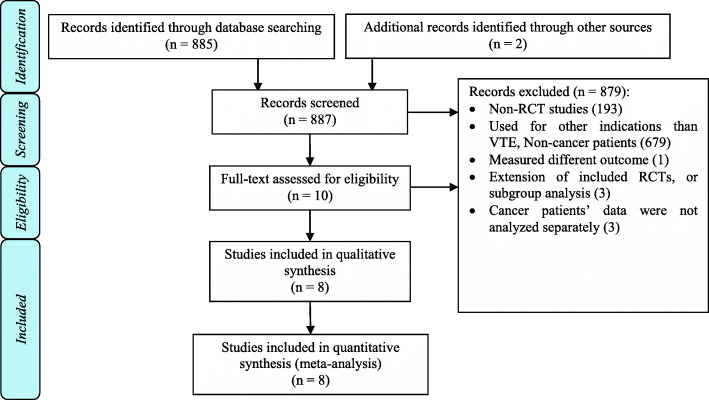
PRISMA flow diagram of the included trials

The literature search yielded a total of 887 publications. All studies matching the inclusion criteria were reviewed and screened for inclusion, and any duplicates or discrepancies were resolved by consensus. A total of 879 articles were excluded based on the date of publication, the language of publication other than English, and non-RCT design. Only eight studies (four regarding thromboprophylaxis and four regarding CA-VTE treatment), including a total of 5360 cancer patients, met the inclusion criteria (Fig. 1) [7, 12, 13, 15–17, 24, 25]. Apixaban for CA-VTE treatment was evaluated in two studies and for thromboprophylaxis in one study [7, 13, 15]; rivaroxaban for CA-VTE treatment was evaluated in two studies and for thromboprophylaxis in one study [12, 16, 24]; edoxaban for CA-VTE treatment was evaluated in one study [17]; and betrixaban for thromboprophylaxis was evaluated in one study [25]. All the included studies were RCTs except for the APEX trial, which reported data on cancer patients in a post-hoc analysis [14], and the MAGELLAN trial, in which data for cancer patients were pooled from a published meta-analysis [26]. In all included studies, VTE and VTE recurrence were confirmed by using compression ultrasonography or *computerized tomography* (*CT*) venography to confirm DVT and CT pulmonary angiography or ventilation/perfusion lung scan (VQ scan) to confirm PE. The characteristics of the included studies were summarized in Table 1. The quality of the studies was presented in Supplementary Fig. S1, while the funnel plots for efficacy and safety outcomes were reported in Supplementary Figs. S2-S10.

**Table 1:** Characteristics of studies included in the meta-analysis

Study			Age (years) mean ± SD	Male sex n (%)	DOACs drug	Comparator	Follow-up (months)	Cancer patients (%)	Main efficacy outcome	Main safety outcome	Phase	Study design
Type	Name	Year										
**Thromboprophylaxis**	MAGELLAN [24]	2013	NR	NR	Rivaroxaban	Enoxaparin	3 months	7.3%	Composite of asymptomatic proximal DVT, symptomatic proximal or distal DVT, symptomatic nonfatal PE, and death from VTE	Clinically relevant bleeding	Phase 3	Subgroup analysis
	APEX [14]	2016	76.8 ± 9.3	240 (48.1)	Betrixaban	Enoxaparin	1 month	12.8%	Composite of asymptomatic proximal DVT, symptomatic proximal or distal DVT, symptomatic nonfatal PE, or fatal PE	Major bleeding	Phase 3	Subgroup analysis
	CASSINI [12]	2019	63^a^	222 (52.9)	Rivaroxaban	Placebo	6 months	100%	Composite of symptomatic or asymptomatic proximal DVT in a lower limb, symptomatic DVT in an upper limb or distal DVT in a lower limb, symptomatic or incidental PE, and death from VTE	Major bleeding	Phase 3	RCT
	AVERT [13]	2019	61.2 ± 12.4	121 (41.6)	Apixaban	Placebo	6 months	100%	VTE	Major bleeding	Phase 3	RCT
**Treatment**	SELECT-D [16]	2018	67^a^	116 (57)	Rivaroxaban	Dalteparin	24 months	100%	Recurrent VTE	Major bleeding	Pilot	RCT
	Hokusai VTE Cancer [17]	2018	64.3 ± 11	277 (53.1)	Edoxaban	Dalteparin	12 months	100%	Recurrent VTE	Major bleeding	Phase 3	RCT
	ADAM VTE [7]	2019	64.4 ± 11.3	72 (48)	Apixaban	Dalteparin	6 months	100%	Recurrent VTE	Major bleeding	Phase 3	RCT
	Caravaggio [15]	2020	67.2 ± 11.3	292 (50.7)	Apixaban	Dalteparin	7 months	100%	Recurrent VTE	Major bleeding	Phase 3	RCT

Abbreviations: *DOAC* direct oral anticoagulant, *DVT* deep vein thrombosis. *PE* pulmonary embolism, *VTE* venous thromboembolism, *RCT* randomized clinical trial

^a^ Median age

### Thromboprophylaxis results

#### Efficacy outcomes

##### VTE events

**Fig. 2:**
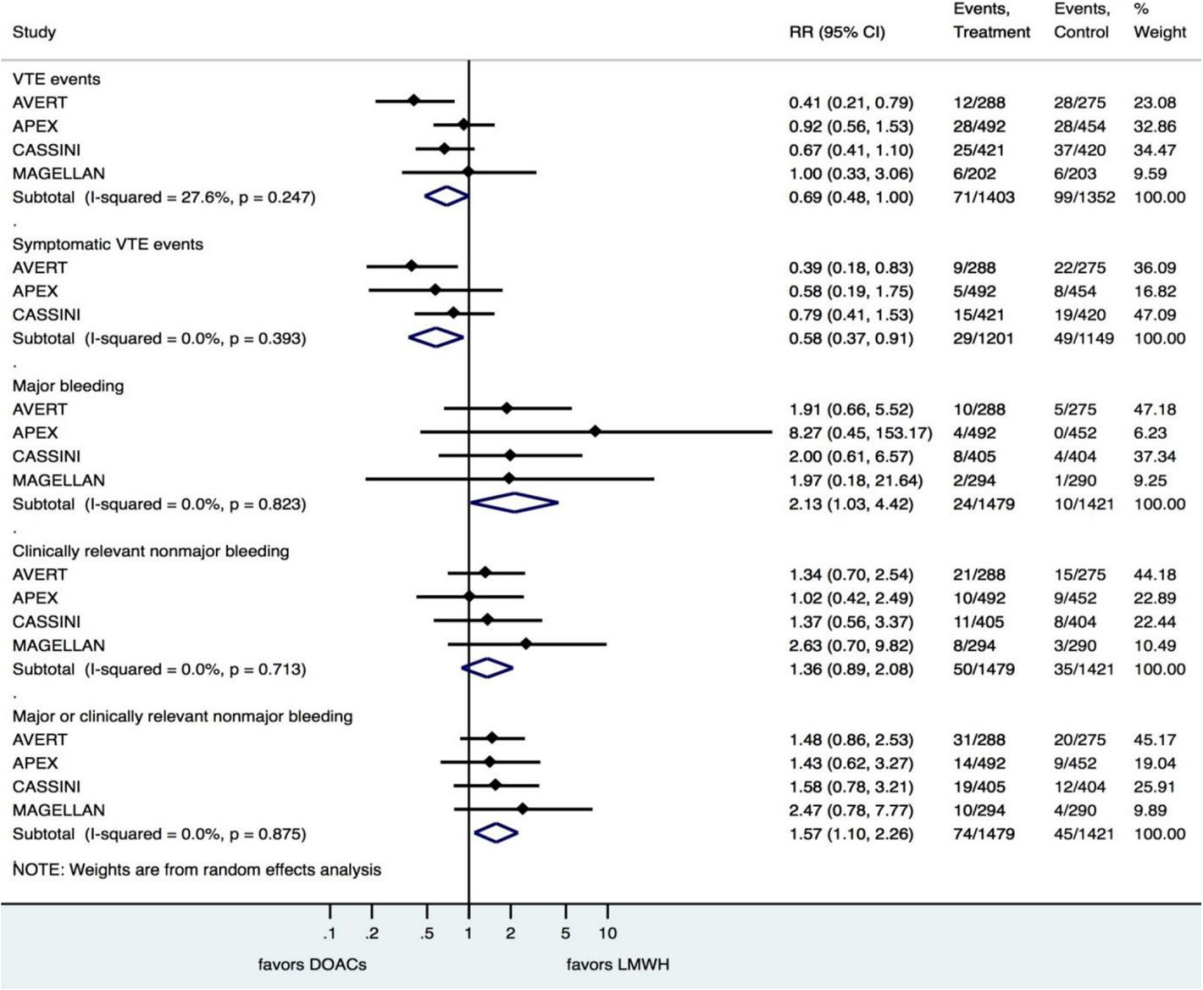
Thromboprophylaxis results of the meta-analysis

There was no difference between DOACs and LMWH or placebo with regard to the occurrence of VTE events (RR = 0.69; 95%CI 0.48, 1.00) (Fig. 2).

##### Symptomatic VTE events

The use of DOACs was associated with a 42% reduction in symptomatic VTE events when compared with LMWH or placebo (RR = 0.58; 95%CI 0.37, 0.91; number needed to treat [NNT] = 45), with no significant heterogeneity (I^2^ = 0%) (Fig. 2).

#### Safety outcomes

##### Major bleeding

The use of DOACs was associated with an approximately two-fold increase in the risk of major bleeding (RR = 2.13; 95%CI 1.03, 4.42; number needed to harm [NNH] = 111), with no significant heterogeneity (I^2^ = 0%) (Fig. 2).

##### CRNMB

There was no difference between DOACs and LMWH or placebo in terms of CRNMB (RR = 1.36; 95%CI 0.89, 2.08) (Fig. 2).

##### Major bleeding or CRNMB

The use of DOACs was associated with an incremental risk of major bleeding or CRNMB when compared with LMWH or placebo (RR = 1.57; 95%CI 1.10, 2.26; NNH = 56), with no significant heterogeneity (I^2^ = 0%) (Fig. 2).

### Treatment results

#### Efficacy outcomes

##### VTE recurrence

**Fig. 3:**
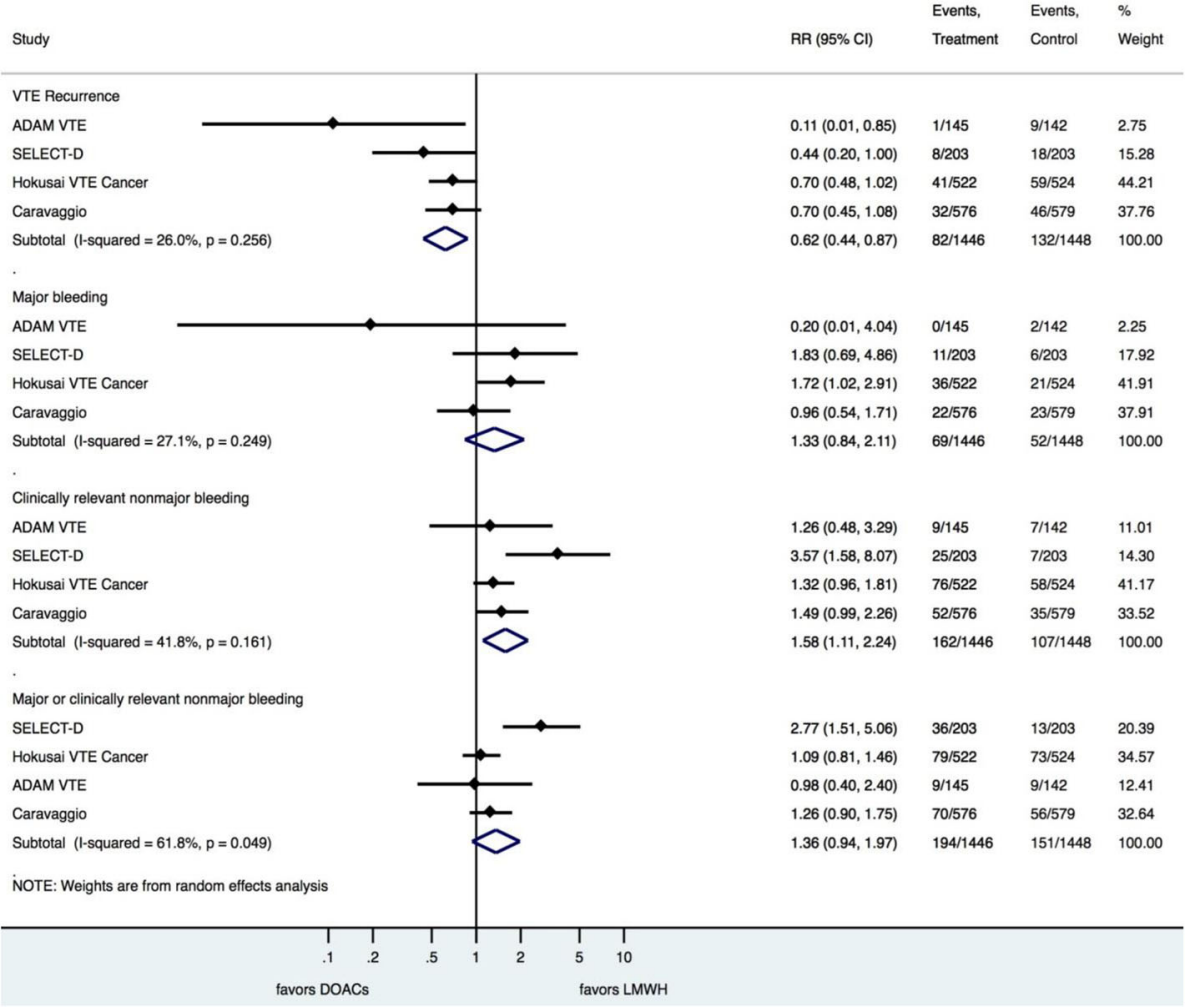
Treatment results of the meta-analysis

The use of DOACs was associated with a 38% reduction in VTE recurrence as compared with LMWH (RR = 0.62; 95%CI 0.44, 0.87; NNT = 29), with no significant heterogeneity (I^2^ = 26%; *p* = 0.256) (Fig. 3).

#### Safety outcomes

##### Major bleeding

There was no difference between the use of DOACs or LMWH with regard to major bleeding events (RR = 1.33; 95%CI 0.84, 2.11) (Fig. 3).

##### CRNMB

The rate of CRNMB was significantly higher with the use of DOACs compared with LMWH (RR = 1.58; 95%CI 1.11, 2.24; NNH = 26), with no significant heterogeneity (I^2^ = 41.8%; *p* = 0.161) (Fig. 3).

##### Major bleeding or CRNMB

There was no difference between the use of DOACs or LMWH with regard to major bleeding or CRNMB (RR = 1.36; 95%CI 0.94, 1.97) (Fig. 3).

### Summary of guideline recommendations

The available guidelines provide controversial recommendations on CA-VTE treatment and prophylaxis in patients with cancer (Table 2). The 2018 guidelines of the International Society on Thrombosis and Haemostasis (ISTH) suggest DOACs for CA-VTE treatment if the bleeding risk is low and there is no risk of drug-drug interactions with anticoagulation, while LMWH is indicated as an alternative option or if there is a high-risk of bleeding or significant drug-drug interactions with anticoagulation. Rivaroxaban or edoxaban is considered as an alternative therapy to LMWH in patients with high bleeding risk with no significant drug-drug interaction [27]. The 2019 European Society of Cardiology (ESC) guidelines recommend LMWH as the first-line option for CA-VTE treatment, and edoxaban or rivaroxaban can be used as alternatives in patients without gastrointestinal (GI) cancer [10]. The 2019 ASCO guidelines recommend the use of LMWH, unfractionated heparin (UFH), fondaparinux, or rivaroxaban for initial CA-VTE treatment, while LMWH, edoxaban, or rivaroxaban is recommended over warfarin for long-term anticoagulation for up to 6 months, with no preference of one over the other beyond the first 6 months [20]. The 2020 National Comprehensive Cancer Network (NCCN) guidelines provide their recommendations on the basis of cancer site. LMWH is recommended as first-line treatment in patients with gastric or gastroesophageal lesions, while apixaban, edoxaban, or rivaroxaban are recommended for patients with other types of cancer [28]. The 2020 ITAC guidelines recommend LMWH if CrCl greater than 30 mL/min; rivaroxaban or edoxaban can be used in these patients if they do not have a high risk of GI or genitourinary bleeding. Fondaparinux is indicated as an alternative option to LMWH, rivaroxaban or edoxaban with the advantage of easier use as compared to UFH. Whereas UFH is indicated in case of contraindication to LMWH, rivaroxaban or edoxaban [21]. In the 2021 ASH guidelines, only rivaroxaban and apixaban were recommended for the initial treatment of CA-VTE, while edoxaban was added to the mentioned DOACs for the short-term treatment of CA-VTE over LMWH in patients with normal renal function. While for Long-term anticoagulation treatment of CA-VTE, the ASH recommended using DOAC, without specifying agents, or LMWH. Nonetheless, the guideline emphasized the cautious use of DOACs in patients with GI cancer [19]. Lastly, the latest ACCP guidelines (2021) now recommend the use of DOACs as a first-line option for CA-VTE treatment over LMWH [22].

**Table 2:** Summary of guideline recommendations for CA-VTE treatment and thromboprophylaxis in patients with cancer

Guideline	Year	CA-VTE Treatment		Thromboprophylaxis
		Preferred or first-line option	Alternative or second-line options	
**ISTH** [27]	2018	*If low risk of bleeding and no drug–drug interactions:* **DOACs**	**LMWH**	NA
**ESC** [10]	2019	**LMWH**	**Edoxaban or rivaroxaban**	NA
**ASCO** [20]	2019	*Initial treatment:* **LMWH, UFH, fondaparinux, or rivaroxaban** *Up to six months:* **LMWH, edoxaban, or rivaroxaban** *Long term anticoagulation:* **LMWH, DOAC, or VKA**	*If unable to obtain LMWH, edoxaban, or rivaroxaban:* **VKA**	*High-risk ambulatory patients (Khorana score ≥ 2):* **LMWH, apixaban, or rivaroxaban**
**NCCN** [28]	2020	*Patients without gastric or gastrointestinal lesions:* **Apixaban, rivaroxaban, or edoxaban** *Patients with gastric or gastrointestinal lesions:* **Dalteparin or enoxaparin**	Dabigatran (with LMWH or UFH for at least 5 days), fondaparinux, UFH, or warfarin	*High risk ambulatory patients (Khorana score ≥ 2)*: **Apixaban or rivaroxaban**
**ITAC** [21]	2020	*Initial treatment and for up to six months:* • *if CrCl ≥ 30 mL/min:* **LMWH** • *if CrCl ≥ 30 mL/min, patient has low risk of GI or genitourinary bleeding, and no drug–drug interactions:* **Edoxaban, or rivaroxaban**	*If no contraindications:* **Fondaparinux** *If patient has contraindication for LMWH or DOACs:* **UFH**	*If hospitalized with reduced mobility and CrCl ≥ 30 mL/min:* **LMWH or fondaparinux**, or **UFH** *For patients with pancreatic cancer or Khorana score ≥ 2 while receiving systemic anticancer therapy at intermediate-to-high risk of VTE and not actively, or at a high-risk of, bleeding:* **Rivaroxaban, apixaban, or LMWH** *Immunomodulatory drugs combined with steroids or other systemic anticancer therapies:* **VKA (at low or therapeutic dose), LMWH, or low-dose aspirin**
**ASH [19]**	2021	*Initial treatment:* **LMWH** *Up to six months:* **DOACs** *Long term anticoagulation:* **DOACs or LMWH**	*If patient has contraindication for LMWH or DOACs:* **UFH**	*If hospitalized:* **LMWH** *High risk ambulatory patients receiving systemic therapy:* **Rivaroxaban or apixaban**
**CHEST [22]**	2021	**DOACs**	**LMWH**	NA

Abbreviations: *CA-VTE* cancer-associated VTE, *DOACs* direct oral anticoagulants, *LMWH* low-molecular-weight heparin, *UFH* unfractionated heparin, *VKA* Vitamin K Antagonist. *ISTH* International Society on Thrombosis and Haemostasis, *ESC* European Society of Cardiology, *ASCO* American Society of Clinical Oncology, *NCCN* National Comprehensive Cancer Network, *ITAC* International Initiative on Thrombosis and Cancer, *CHEST* American College of Chest Physicians, *NA* no specific recommendation for DOACs is available

For thromboprophylaxis, the ASCO, NCCN, ITAC, and ASH guidelines recommend the use of thromboprophylaxis with apixaban or rivaroxaban (or LMWH in the ASCO) for moderate to high-risk ambulatory cancer patients with a Khorana score of two or higher [19–21, 28]; the ITAC guideline extend this recommendation to patients with intermediate-risk or pancreatic cancer, but limit it to patients with no active, or at a high risk of, bleeding [21]. Most of the guidelines’ recommendations for CA-VTE treatment and thromboprophylaxis were summarized in Table 2.

## Discussion

This systematic review and meta-analysis evaluated the efficacy and safety of using DOACs compared with LMWH or placebo for thromboprophylaxis and LMWH for CA-VTE treatment. By pooling the data from RCTs and post-hoc analyses of RCTs, more than 5300 patients from eight trials were analyzed. The use of DOACs was associated with about 40% lower risk of VTE recurrence and symptomatic VTE when used for CA-VTE treatment and thromboprophylaxis, respectively. However, when DOACs were used for thromboprophylaxis, the risk of major bleeding was twice higher when compared with placebo or LMWH. When thromboprophylaxis data were analyzed separately based on the comparator (i.e., placebo or LMWH), no difference was found between DOACs and LMWH in both efficacy and safety outcomes (Supplementary Fig. S11), while in studies that use placebo as a comparator, DOACs showed a significant reduction in VTE events with no difference in the other efficacy and safety outcomes (Supplementary Fig. S12). On the other hand, when DOACs were used for CA-VTE treatment, the risk of major bleeding was not significantly higher than that for LMWH.

The use of DOACs for thromboprophylaxis in patients with cancer reduced the risk of symptomatic VTE events (RR = 0.58; 95%CI 0.37, 0.91). This result is in contrast to a previous meta-analysis, in which patients with cancer who received DOACs for thromboprophylaxis showed a nonsignificant reduction in the risk of symptomatic VTE (RR = 0.57; 95%CI 0.29, 1.12) [29]. This discrepancy is mainly due to the fact that the previous meta-analysis included only the AVERT and CASSINI trials. In the present study, the efficacy of DOACs was compared against placebo in patients who mostly had a Khorana score of two or higher [12, 13, 29]. However, the AVERT trial utilized a modified Khorana risk score, which led to the inclusion of patients with more types of cancer [13]. Even though the Khorana scores for patients included in the subgroup analysis of the MAGELLAN and APEX trials were not reported [14, 24], the addition of data from their subgroup analyses provided significant evidence supporting the use of DOACs for thromboprophylaxis to reduce the risk of VTE in patients with cancer. The majority of those trials included high-risk patients with cancer, including GI, lung, pancreatic, and metastatic cancer. In contrast, a limited number of patients with brain malignancies were included only in the AVERT trial, which may weaken the evidence supporting the use of DOACs for thromboprophylaxis in this population.

Similar to previous systematic reviews and meta-analyses that assessed the use of DOACs for CA-VTE treatment [30–32], our meta-analysis showed that DOACs were associated with a 38% risk reduction of VTE recurrence compared with LMWH. This rate was comparable to the rates reported in previous meta-analyses, which ranged from 32 to 55% [30–32]. Mulder et al. found the risk of VTE recurrence in patients using DOACs to be nonsignificantly lower than that for the LMWH group (RR = 0.68; 95%CI 0.39, 1.17) [30]. However, the most recent meta-analysis reported DOACs to be associated with a significantly lower risk of VTE recurrence compared with LMWH in patients with cancer [32], even though it did not include data from the Caravaggio trial, one of the largest trials that demonstrated the efficacy of DOACs in preventing VTE recurrence in patients with CA-VTE. The overall risk reduction of VTE recurrence observed in our meta-analysis was mainly driven by the results from the ADAM VTE trial, in which the VTE recurrence was a secondary outcome that was barely observed [7]. Moreover, the ADAM VTE was an open-label RCT that included a small sample of patients (*n* = 300) compared with the Caravaggio trial (*n* = 1155); however, the percentage of patients with upper GI cancer was similar in both trials [7, 16].

In this meta-analysis, the use of DOACs for CA-VTE treatment or thromboprophylaxis was associated with a higher risk of bleeding than LMWH or placebo. The risk of major bleeding was nonsignificantly higher with DOACs when used for CA-VTE treatment (RR = 1.33, 95%CI 0.84, 2.11), while it was twice higher when DOACs were used for thromboprophylaxis (RR = 2.13, 95%CI 1.03, 4.42). Since the definition of major bleeding was almost consistent across the studies, the differences in the risk of major bleeding can be attributed to the heterogeneity of the cancer patient population. Compared with other trials, betrixaban in the APEX trial and rivaroxaban in the CASSINI trial were associated with a higher risk of major bleeding [12, 14]. Nonetheless, the number of patients with GI and metastatic malignancies in the APEX and CASSINI trials was higher than in the AVERT trial, which might explain the increase in bleeding risk [12–14]. Here, the risk of CRNMB did not differ between the DOACs and LMWH or placebo when used for thromboprophylaxis. However, the risk of CRNMB was significantly higher among patients receiving DOACs for CA-VTE treatment. This increase was mainly driven by the SELECT-D trial, as it reported a higher risk of CRNMB in patients receiving rivaroxaban than in those on dalteparin [16]. It is also worth mentioning that the SELECT-D trial subsequently excluded patients with GI malignancies due to the increased risk of bleeding [16].

Until recently, most of the previous guidelines recommended LMWH as the first-line therapy for CA-VTE treatment over VKAs and DOACs [9, 33, 34]. This was due to insufficient evidence that supported the efficacy and safety of DOACs in patients with cancer, especially that DOAC approval trials only included a small proportion of patients with a history of active cancer [34]. However, numerous RCTs and observational studies have been published recently that used DOACs for the treatment or prevention of CA-VTE [7, 12, 13, 15–17, 25]. Also, several meta-analyses supported the lower risk of VTE recurrence and bleeding with DOACs as compared with LMWH and VKAs [30–32]. This body of evidence from RCTs and meta-analyses prompted some of the large organizations to add DOACs as a viable option for CA-VTE treatment or thromboprophylaxis in their latest guidelines [20, 21]. The ACCP, ASCO, ITAC, NCCN, and ASH have recently updated their guidelines to recommend the use of edoxaban, apixaban, or rivaroxaban as initial therapy for CA-VTE treatment in patients without gastric lesions or GI malignancies due to the increased risk of bleeding in these patients [19–22, 28]. Clinical guidelines do not recommend the routine use of pharmacological thromboprophylaxis in all patients with cancer [9, 10, 20, 21, 28]. This is mainly due to the relatively low risk of VTE in ambulatory patients with cancer and the increased risk of bleeding with the use of anticoagulants [20, 21]. The use of validated VTE risk prediction tools such as the Khorana VTE risk assessment tool can help identify patients with cancer receiving chemotherapy who are at greater risk for VTE [34]. The use of those tools prompted the development of newer guidelines supporting the use of DOACs in high-risk patients who are not at increased risk of bleeding. Thus, the current guidelines recommend the use of rivaroxaban and apixaban in patients at intermediate-to-high-risk of VTE, identified by cancer type (i.e., pancreatic) or a Khorana score of two or higher in the absence of active bleeding or not at high-risk of bleeding [20, 21]. The convenience of oral administration of DOACs provides an easier, yet effective, therapeutic alternative for subcutaneous anticoagulation, which may increase patient adherence. However, caution should be warranted with the use of DOACs in patients with high-risk of bleeding and brain malignancies due to limited safety data.

To our knowledge, this is the first systematic review and meta-analysis that assessed the efficacy and safety of DOACs for both primary and secondary prevention of CA-VTE. Nevertheless, as any other meta-analysis, it is not free of limitations. There was a variation among the included trials in terms of patient population and follow-up duration, but this does not seem to have impacted the results as no significant heterogeneity was observed in the analysis. Although we included only high-quality RCTs to assess the efficacy and safety of DOACs for CA-VTE treatment, limited RCTs were available to assess the efficacy of DOACs for thromboprophylaxis. Therefore, data from subgroup or post-hoc analyses of large RCTs were included and the heterogeneity between these studies was low. The LMWH comparator included in our analysis was limited to dalteparin, as in all previous trials starting with the CLOT trial [6]. Also, some included studies excluded patients with hematological, brain, and metastatic brain tumors, so caution is needed when generalizing our results to those populations. Finally, this analysis did not include all DOACs for both thromboprophylaxis and treatment of CA-VTE, since there were no studies that assessed the use of edoxaban for thromboprophylaxis, betrixaban for treatment, or dabigatran for both.

## Conclusions

This meta-analysis highlighted the efficacy and safety of DOACs for thromboprophylaxis and treatment of CA-VTE. DOACs demonstrated a lower risk of VTE recurrence than LMWH and a lower risk of symptomatic VTE than LMWH or placebo. However, the risk of bleeding remains an important concern. Clinical decisions on the use of DOACs for CA-VTE treatment or prophylaxis should be based on individual assessment of the patient’s risk for VTE and bleeding, using validated risk assessment tools. The findings can provide an additional insight into the development of future guidelines and protocols aimed to optimize anticoagulation therapy in patients with cancer.

## Supplementary Information

**Additional file 1 Table S1.** Definitions of the outcomes for included studies. **Fig. S1.** Quality assessment of included randomized controlled trials. **Fig. S2.** Funnel plot for the VTE events outcome in the thromboprophylaxis studies. F**ig. S3.** Funnel plot for the symptomatic VTE events outcome in the thromboprophylaxis studies. **Fig. S4.** Funnel plot for the major bleeding events outcome in the thromboprophylaxis studies. **Fig. S5.** Funnel plot for the clinically relevant nonmajor bleeding events outcome in the thromboprophylaxis studies. **Fig. S6.** Funnel plot for the major or clinically relevant nonmajor bleeding events outcome in the thromboprophylaxis studies. **Fig. S7**. Funnel plot for the VTE recurrence outcome in the treatment studies. **Fig. S8.** Funnel plot for the major bleeding events outcome in the treatment studies. **Fig. S9.** Funnel plot for the clinically relevant nonmajor bleeding events outcome in the treatment studies. **Fig. S10.** Funnel plot for the major or clinically relevant nonmajor bleeding events outcome in the treatment studies. **Fig. S11**. Thromboprophylaxis results (DOACs vs. LMWH). **Fig. S12.** Thromboprophylaxis results (DOACs vs. placebo).

## Data Availability

All data generated or analyzed during this study are included in this published article [and its supplementary information files].

## References

[1] Khorana AA, Francis CW, Culakova E, Kuderer NM, Lyman GH. Thromboembolism is a leading cause of death in cancer patients receiving outpatient chemotherapy. J Thromb Haemost. 2007;5(3). 10.1111/j.1538-7836.2007.02374.x. 10.1111/j.1538-7836.2007.02374.x 17319909

[2] Giustozzi M, Curcio A, Weijs B, Field TS, Sudikas S, Katholing A, et al. Variation in the association between antineoplastic therapies and venous thromboembolism in patients with active cancer. Thromb Haemost. 2020;120(5). 10.1055/s-0040-1709527. 10.1055/s-0040-1709527 32369855

[3] Elyamany G, Alzahrani AM, Bukhary E (2014). Cancer-associated thrombosis: an overview. Clin Med Insights Oncol.

[4] Prandoni P, Lensing AW, Piccioli A, Bernardi E, Simioni P, Girolami B, et al. Recurrent venous thromboembolism and bleeding complications during anticoagulant treatment in patients with cancer and venous thrombosis. Blood. 2002;100(10). 10.1182/blood-2002-01-0108. 10.1182/blood-2002-01-0108 12393647

[5] Lee AY, Kamphuisen PW, Meyer G, Bauersachs R, Janas MS, Jarner MF, et al. Tinzaparin vs warfarin for treatment of acute venous thromboembolism in patients with active cancer: a randomized clinical trial. JAMA. 2015;314(7). 10.1001/jama.2015.9243. 10.1001/jama.2015.9243 26284719

[6] Lee AY, Levine MN, Baker RI, Bowden C, Kakkar AK, Prins M, et al. Low-molecular-weight heparin versus a coumarin for the prevention of recurrent venous thromboembolism in patients with cancer. N Engl J Med. 2003;349(2). 10.1056/NEJMoa025313. 10.1056/NEJMoa025313 12853587

[7] McBane RD 2nd, Wysokinski WE, Le-Rademacher JG, Zemla T, Ashrani A, Tafur A, et al. Apixaban and dalteparin in active malignancy-associated venous thromboembolism: The ADAM VTE trial. J Thromb Haemost. 2020;18(2). 10.1111/jth.14662. 10.1111/jth.14662 31630479

[8] Seaman S, Nelson A, Noble S. Cancer-associated thrombosis, low-molecular-weight heparin, and the patient experience: a qualitative study. Patient Prefer Adherence. 2014;8. 10.2147/PPA.S58595. 10.2147/PPA.S58595 PMC3986276 24748774

[9] Kearon C, Akl EA, Ornelas J, Blaivas A, Jimenez D, Bounameaux H, et al. Antithrombotic therapy for VTE disease: CHEST guideline and expert panel report. Chest. 2016;149(2). 10.1016/j.chest.2015.11.026. 10.1016/j.chest.2015.11.026 26867832

[10] Konstantinides SV, Meyer G, Becattini C, Bueno H, Geersing G-J, Harjola V-P, et al. ESC guidelines for the diagnosis and management of acute pulmonary embolism developed in collaboration with the European Respiratory Society (ERS). Eur Heart J. 2019, 2020;41(4). 10.1093/eurheartj/ehz405. 10.1093/eurheartj/ehz405 31504429

[11] Witt DM, Nieuwlaat R, Clark NP, Ansell J, Holbrook A, Skov J, et al. American Society of Hematology 2018 guidelines for management of venous thromboembolism: optimal management of anticoagulation therapy. Blood Adv. 2018;2(22). 10.1182/bloodadvances.2018024893. 10.1182/bloodadvances.2018024893 PMC6258922 30482765

[12] Khorana AA, Soff GA, Kakkar AK, Vadhan-Raj S, Riess H, Wun T, et al. Rivaroxaban for thromboprophylaxis in high-risk ambulatory patients with cancer. N Engl J Med. 2019;380(8). 10.1056/NEJMoa1814630. 10.1056/NEJMoa1814630 30786186

[13] Carrier M, Abou-Nassar K, Mallick R, Tagalakis V, Shivakumar S, Schattner A, et al. Apixaban to prevent venous thromboembolism in patients with cancer. N Engl J Med. 2019;380(8). 10.1056/NEJMoa1814468. 10.1056/NEJMoa1814468 30511879

[14] Ageno W, Lopes RD, Yee MK, Hernandez A, Hull R, Goldhaber SZ, et al. Extended prophylaxis of venous thromboembolism with betrixaban in acutely ill medical patients with and without cancer: insights from the APEX trial. J Thromb Thrombolysis. 2020;49(2). 10.1007/s11239-019-01943-5. 10.1007/s11239-019-01943-5 31493287

[15] Agnelli G, Becattini C, Meyer G, Muñoz A, Huisman MV, Connors JM, et al. Apixaban for the treatment of venous thromboembolism associated with cancer. N Engl J Med. 2020;382(17). 10.1056/NEJMoa1915103. 10.1056/NEJMoa1915103 32223112

[16] Young AM, Marshall A, Thirlwall J, Chapman O, Lokare A, Hill C, et al. Comparison of an oral factor Xa inhibitor with low molecular weight heparin in patients with cancer with venous thromboembolism: results of a randomized trial (SELECT-D). J Clin Oncol. 2018. 10.1200/JCO.2018.78.8034. 10.1200/JCO.2018.78.8034 29746227

[17] Raskob GE, van Es N, Verhamme P, Carrier M, Di Nisio M, Garcia D, et al. Edoxaban for the treatment of cancer-associated venous thromboembolism. N Engl J Med. 2018;378(7). 10.1056/NEJMoa1711948. 10.1056/NEJMoa1711948 29231094

[18] Khorana AA, Francis CW, Kuderer NM, Carrier M, Ortel TL, Wun T, et al. Dalteparin thromboprophylaxis in cancer patients at high risk for venous thromboembolism: a randomized trial. Thromb Res. 2017;151. 10.1016/j.thromres.2017.01.009. 10.1016/j.thromres.2017.01.009 28139259

[19] Lyman GH, Carrier M, Ay C, Di Nisio M, Hicks LK, Khorana AA, et al. American society of hematology 2021 guidelines for management of venous thromboembolism: prevention and treatment in patients with cancer. Blood Adv. 2021;5(4). 10.1182/bloodadvances.2020003442. 10.1182/bloodadvances.2020003442 PMC7903232 33570602

[20] Key NS, Khorana AA, Kuderer NM, Bohlke K, Lee AY, Arcelus JI, et al. Venous thromboembolism prophylaxis and treatment in patients with cancer: ASCO clinical practice guideline update. J Clin Oncol. 2020;38(5). 10.1200/JCO.19.01461. 10.1200/JCO.19.01461 31381464

[21] Farge D, Frere C, Connors JM, Ay C, Khorana AA, Munoz A, et al. International clinical practice guidelines for the treatment and prophylaxis of venous thromboembolism in patients with cancer. Lancet Oncol. 2019, 2019;20(10). 10.1016/s1470-2045(19)30336-5. 10.1016/S1470-2045(19)30336-5 31492632

[22] Stevens SM, Woller SC, Baumann Kreuziger L, Bounameaux H, Doerschug K, Geersing G-J, et al. Antithrombotic therapy for VTE disease: second update of the CHEST guideline and expert panel report - executive summary. Chest. 2021. 10.1016/j.chest.2021.07.056. 10.1016/j.chest.2021.07.056 34352279

[23] Higgins JPT, Thomas J, Chandler J, Cumpston M, Li T, Page MJ, Welch VA (editors). Cochrane Handbook for Systematic Reviews of Interventions. 2nd Edition. Chichester (UK): Wiley; 2019.

[24] Cohen AT, Spiro TE, Büller HR, Haskell L, Hu D, Hull R, et al. Rivaroxaban for thromboprophylaxis in acutely ill medical patients. N Engl J Med. 2013;368. 10.1056/NEJMoa1111096. 10.1056/NEJMoa1111096 23388003

[25] Cohen AT, Harrington RA, Goldhaber SZ, Hull RD, Wiens BL, Gold A, et al. Extended thromboprophylaxis with betrixaban in acutely ill medical patients. N Engl J Med. 2016;375(6). 10.1056/NEJMoa1601747. 10.1056/NEJMoa1601747 27232649

[26] Vaidya SR, Gupta S, Devarapally SR. Treatment of cancer-associated venous thromboembolism by new oral anticoagulants: a meta-analysis. J Xiangya Med. 2017. 10.21037/jxym.2017.07.04.

[27] Khorana AA, Noble S, Lee AYY, Soff G, Meyer G, O'Connell C, et al. Role of direct oral anticoagulants in the treatment of cancer-associated venous thromboembolism: guidance from the SSC of the ISTH. J Thromb Haemost. 2018;16(9). 10.1111/jth.14219. 10.1111/jth.14219 30027649

[28] Streiff MB, Holmstrom B, Angelini D, Ashrani A, Elshoury A, Fanikos J et al. Cancer-associated venous thromboembolic disease (Version 1. 2020). (2020). https://www.nccn.org/professionals/physician_gls/pdf/vte.pdf Accessed July 27, 2020.

[29] Li A, Kuderer NM, Garcia DA, Khorana AA, Wells PS, Carrier M, et al. Direct oral anticoagulant for the prevention of thrombosis in ambulatory patients with cancer: a systematic review and meta-analysis. J Thromb Haemost. 2019;17(12). 10.1111/jth.14613. 10.1111/jth.14613 31420937

[30] Mulder FI, Bosch F, Young AM, Marshall A, McBane RD, Zemla T, et al. Direct oral anticoagulants for cancer-associated venous thromboembolism: a systematic review and meta-analysis. Blood. 2020. 10.1182/blood.2020005819. 10.1182/blood.2020005819 32396939

[31] Sabatino J, De Rosa S, Polimeni A, Sorrentino S, Indolfi C. Direct oral anticoagulants in patients with active cancer: a systematic review and meta-analysis. JACC CardioOncol. 2020. 10.1016/j.jaccao.2020.06.001. 10.1016/j.jaccao.2020.06.001 PMC8352218 34396250

[32] Sidahmed S, Abdalla A, Kheiri B, Bala A, Salih M, Bachuwa G, et al. Anticoagulants for the treatment of venous thromboembolism in patients with cancer: A comprehensive systematic review, pairwise and network meta-analysis. Crit Rev Oncol Hematol. 2020;152. 10.1016/j.critrevonc.2020.103005. 10.1016/j.critrevonc.2020.103005 32540780

[33] Farge D, Bounameaux H, Brenner B, Cajfinger F, Debourdeau P, Khorana AA, et al. International clinical practice guidelines including guidance for direct oral anticoagulants in the treatment and prophylaxis of venous thromboembolism in patients with cancer. Lancet Oncol. 2016;17(10). 10.1016/s1470-2045(16)30369-2. 10.1016/S1470-2045(16)30369-2 27733271

[34] Lyman GH, Kuderer NM. Clinical practice guidelines for the treatment and prevention of cancer-associated thrombosis. Thromb Res. 2020;191. 10.1016/S0049-3848(20)30402-3. 10.1016/S0049-3848(20)30402-3 32736784

