# Supplementary material for: The use of direct oral anticoagulants for thromboprophylaxis or treatment of cancer-associated venous thromboembolism: a meta-analysis and review of the guidelines
Source: Thromb J. 2021 Oct 30;19:76. doi: 10.1186/s12959-021-00326-2 (PMC8556878; doi:10.1186/s12959-021-00326-2)
Supplement: Supplementary file 1 — Additional file 1 Table S1. Definitions of the outcomes for included studies. Fig. S1. Quality assessment of included randomized controlled trials. Fig. S2. Funnel plot for the VTE events outcome in the thromboprophylaxis studies. Fig. S3. Funnel plot for the symptomatic VTE events outcome in the thromboprophylaxis studies. Fig. S4. Funnel plot for the major bleeding events outcome in the thromboprophylaxis studies. Fig. S5. Funnel plot for the clinically relevant nonmajor bleeding events outcome in the thromboprophylaxis studies. Fig. S6. Funnel plot for the major or clinically relevant nonmajor bleeding events outcome in the thromboprophylaxis studies. Fig. S7. Funnel plot for the VTE recurrence outcome in the treatment studies. Fig. S8. Funnel plot for the major bleeding events outcome in the treatment studies. Fig. S9. Funnel plot for the clinically relevant nonmajor bleeding events outcome in the treatment studies. Fig. S10. Funnel plot for the major or clinically relevant nonmajor bleeding events outcome in the treatment studies. Fig. S11. Thromboprophylaxis results (DOACs vs. LMWH). Fig. S12. Thromboprophylaxis results (DOACs vs. placebo). [file 12959_2021_326_MOESM1_ESM.docx]

| **Table S1.** Definitions of the outcomes for included studies | | | |
| --- | --- | --- | --- |
| **Studies** | | **Outcome** | **Definition** |
| Thromboprophylaxis studies | MAGELLAN | VTE occurrence | Asymptomatic proximal DVT, symptomatic proximal or distal DVT, symptomatic nonfatal PE, or VTE-related death. |
|  |  | Major Bleeding | Bleeding leading to a ≥2 g/dl fall in hemoglobin or a transfusion of ≥2 units of packed RBCs or whole blood, bleeding into a critical site (intracranial, intraspinal, intraocular, retroperitoneal, intra-articular, pericardial, or intramuscular with compartment syndrome), or bleeding leading to death. |
|  |  | CRNMB | Overt bleeding not meeting the criteria for major bleeding but associated with medical intervention, unscheduled contact with a physician, temporary cessation of study treatment or discomfort for the subject such as pain, or impairment of activities of daily life |
|  | APEX | VTE occurrence | Asymptomatic proximal DVT (as detected by ultrasound), symptomatic proximal or distal DVT, non-fatal PE, or VTE-related death. |
|  |  | Major Bleeding | Fatal bleeding, and/or symptomatic bleeding in a critical area or organ, such as intracranial, intraspinal, intraocular, retroperitoneal, intra‐articular or pericardial, or intramuscular with compartment syndrome, and/or bleeding causing a fall in hemoglobin level of ≥2 g/dl, or leading to transfusion of ≥2 units of whole blood or RBCs. |
|  |  | CRNMB | Overt bleeding not meeting criteria for major bleeding but associated with medical intervention, unscheduled contact with a physician, cessation of study treatment, or associated with discomfort for the patient. |
|  | CASSINI | VTE occurrence | Objectively confirmed symptomatic or asymptomatic proximal DVT in a lower limb, symptomatic DVT in an upper limb or distal DVT in a lower limb, symptomatic or incidental PE, and VTE-related death. |
|  |  | Major Bleeding | Fatal bleeding, and/or symptomatic bleeding in a critical area or organ, such as intracranial, intraspinal, intraocular, retroperitoneal, intra‐articular or pericardial, or intramuscular with compartment syndrome, and/or bleeding causing a fall in hemoglobin level of ≥2 g/dl, or leading to transfusion of ≥2 units of whole blood or RBCs. |
|  |  | CRNMB | Any sign or symptom of hemorrhage that does not fit the criteria for the ISTH definition of major bleeding but does meet at least one of the following criteria: requiring medical intervention by a healthcare professional, leading to hospitalization or increased level of care, prompting a face to face evaluation |
|  | AVERT | VTE occurrence | objectively documented major VTE (proximal DVT or PE within the first 180 days (with a window of ±3 days) after randomization |
|  |  | Major Bleeding | overt bleeding that was associated with a decrease in the hemoglobin level of ≥2 g/dl, led to transfusion of ≥2 units of packed RBCs, occurred in a critical site, or contributed to death |
|  |  | CRNMB | Acute, clinically over bleeding episodes that did not meet the criteria for major bleeding episodes but were associated with medical intervention, unscheduled contact with a physician, interruption or discontinuation of the assigned treatment or discomfort or impairment of activities of daily life. |
| Treatment studies | SELECT-D | VTE Recurrence | Recurrent proximal DVT, or recurrent PE (symptomatic or incidental), or fatal PE, or other sites of venous thrombosis (e.g., subclavian vein, hepatic vein, or inferior vena cava) |
|  |  | Major Bleeding | Acute, clinically overt bleeding accompanied by ≥1 of the following findings: a decrease in the hemoglobin level of ≥2 g/dl over a 24-h period, transfusion of ≥2 units of packed RBCs, bleeding at a critical site (including intracranial, intraspinal, intraocular, pericardial, or retroperitoneal bleeding), or fatal bleeding |
|  |  | CRNMB | Acute, clinically overt episodes, such as wound hematoma, bruising, GI bleeding, hemoptysis, hematuria, or epistaxis that did not meet the criteria for major bleeding but were associated with medical intervention, unscheduled contact with a physician, interruption or discontinuation of a study drug, or discomfort or impairment of activities of daily life |
|  | Hokusai VTE Cancer | VTE Recurrence | Symptomatic new DVT or PE, incidental (detected by means of imaging tests performed for other reasons) new DVT, or PE involving segmental or more proximal pulmonary arteries, or fatal PE or unexplained death for which PE could not be ruled out as the cause |
|  |  | Major Bleeding | Overt bleeding that was associated with a decrease in the hemoglobin level of ≥2 g/dl, led to a transfusion of ≥2 units of blood, occurred in a critical site, or contributed to death. |
|  |  | CRNMB | Overt bleeding that did not meet the criteria for major bleeding but was associated with the use of medical intervention, contact with a physician, interruption of the assigned treatment, discomfort, or impairment of activities of daily living. |
|  | ADAM VTE | VTE Recurrence | Any thromboembolic recurrence including DVT, PE, fatal PE, or arterial thromboembolism. A recurrent event was a new filling defect evident on the second study not appreciated on the original images, or when an interval study clearly showed thrombus resolution. An arterial thromboembolism could include myocardial infarction, stroke, transient ischemic attack, or peripheral arterial embolism. |
|  |  | Major Bleeding | Overt bleeding plus a hemoglobin decrease of ≥2 g/dl or transfusion of ≥2 units of packed RBCs, or intracranial, intraspinal/epidural, intraocular, retroperitoneal, pericardial, intra-articular, or intramuscular with compartment syndrome, or fatal bleeding |
|  |  | CRNMB | Overt bleeding not meeting the criteria for major bleeding but associated with medical intervention, an unscheduled contact with the health care team, or temporary anticoagulant cessation |
|  | Caravaggio | VTE Recurrence | Proximal DVT of the lower limbs (symptomatic or incidental), symptomatic DVT of the upper limbs, or PE (symptomatic, incidental, or fatal) occurring during the 6-month trial period |
|  |  | Major Bleeding | Acute clinically overt bleeding associated with ≥1: 1) decrease in the hemoglobin level of at least 2 g/dl; 2) transfusion of ≥2 units of RBCs; 3) bleeding occurring at a critical site (intracranial, intraspinal, intraocular, pericardial, intra-articular, intramuscular with compartment syndrome, or retroperitoneal); 4) bleeding resulting in surgical intervention, or fatal bleeding, all occurring during the trial drug period through 72 h after the last dose was administered |
|  |  | CRNMB | Acute clinically overt bleeding that does not meet the criteria for major bleeding and consists of: 1) any bleeding compromising hemodynamics; 2) spontaneous hematoma >25 cm2, or 100 cm2 if there was a traumatic cause; 3) intramuscular hematoma documented by ultrasonography; 4) epistaxis or gingival bleeding requiring tamponade or other medical intervention or bleeding from venipuncture for >5 min; 5) hematuria that was macroscopic and was spontaneous or lasted for >24 h after invasive procedures; 6) hemoptysis, hematemesis, or spontaneous rectal bleeding requiring endoscopy or other medical intervention; 7) or any other bleeding considered to have clinical consequences for a patient, such as medical intervention, the need for unscheduled contact (visit or telephone call) with a physician, or temporary cessation of a study drug, or associated with pain or impairment of activities of daily life. |
| Abbreviations: DVT: deep vein thrombosis. PE: pulmonary embolism. VTE: venous thromboembolism. CRNMB: clinically relevant non-major bleeding. RBCs: red blood cells. ISTH: International Society on Thrombosis and Haemostasis | | | |


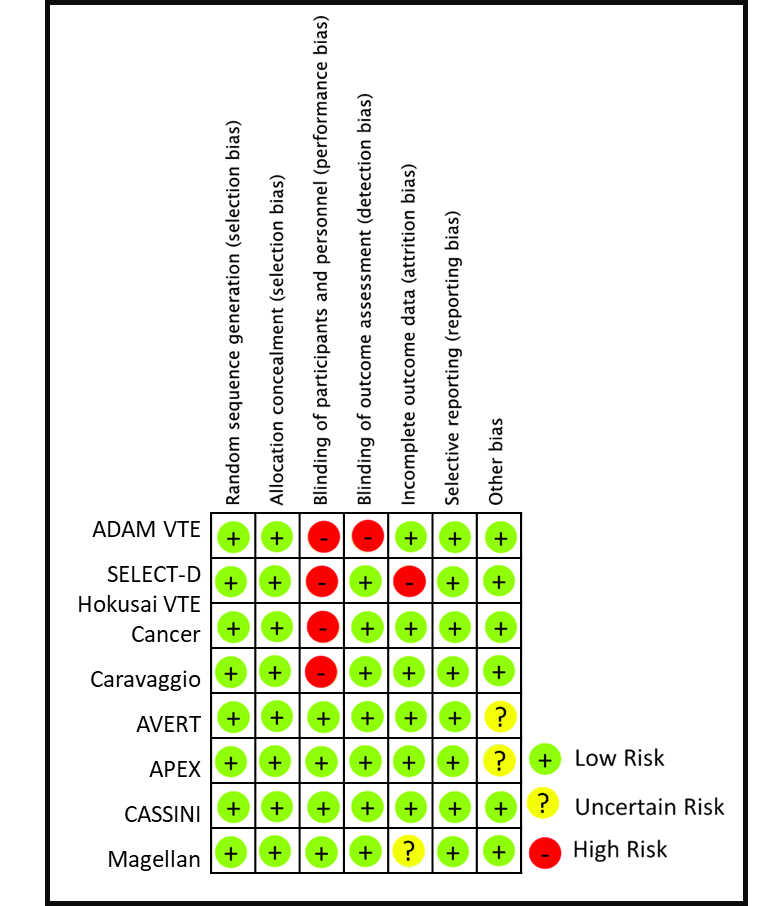


**Figure S1.** Quality assessment of included randomized controlled trials

**Figure S3.** Funnel plot for the symptomatic VTE events outcome in the thromboprophylaxis studies


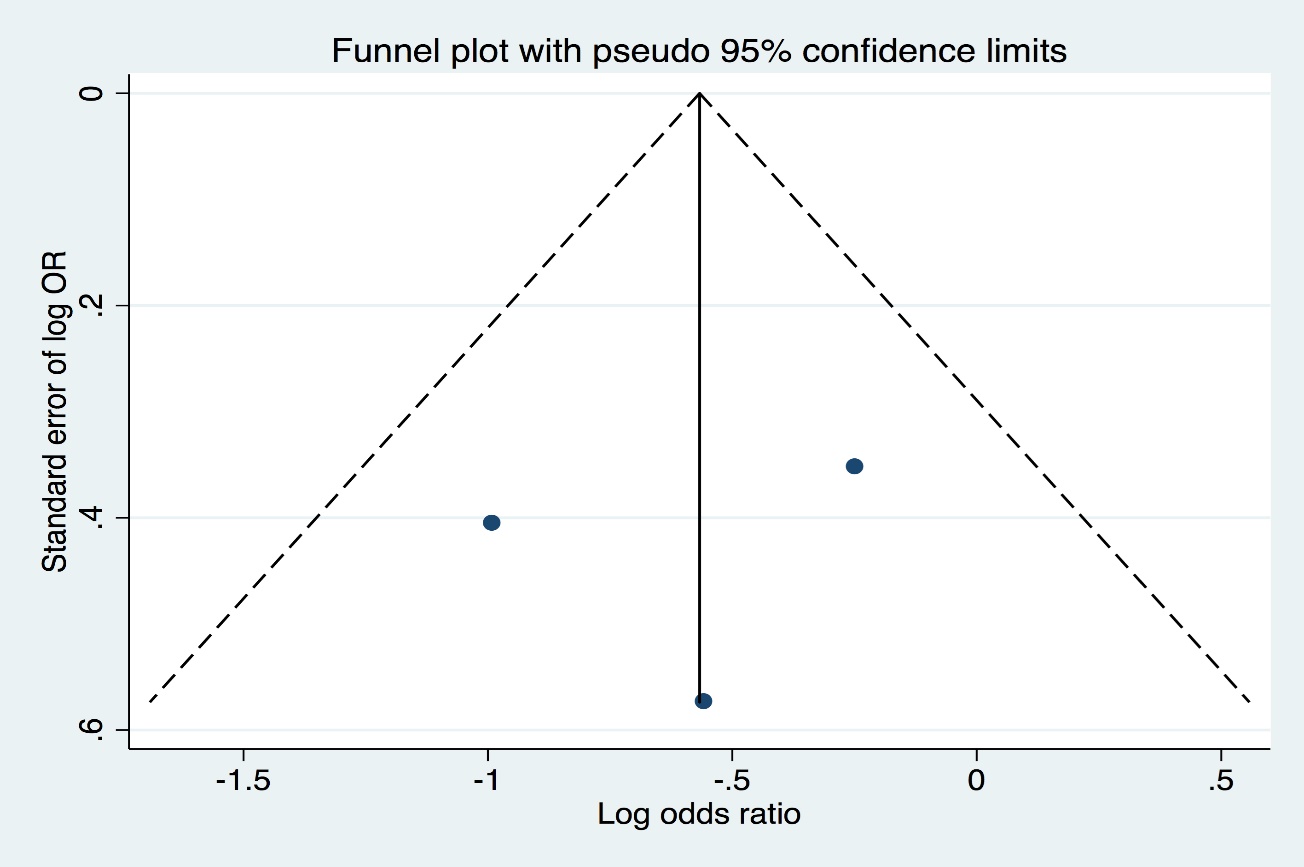

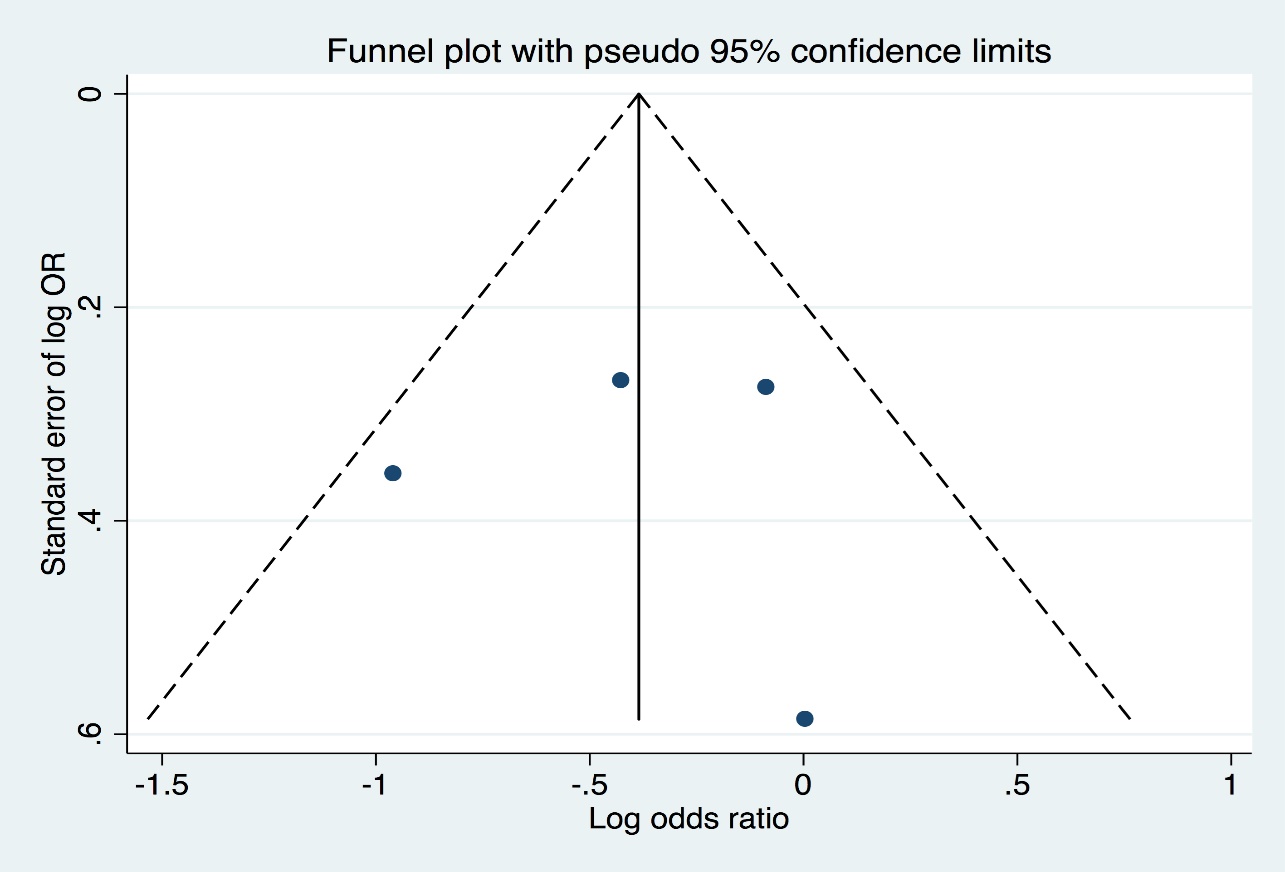


**Figure S2.** Funnel plot for the VTE events outcome in the thromboprophylaxis studies


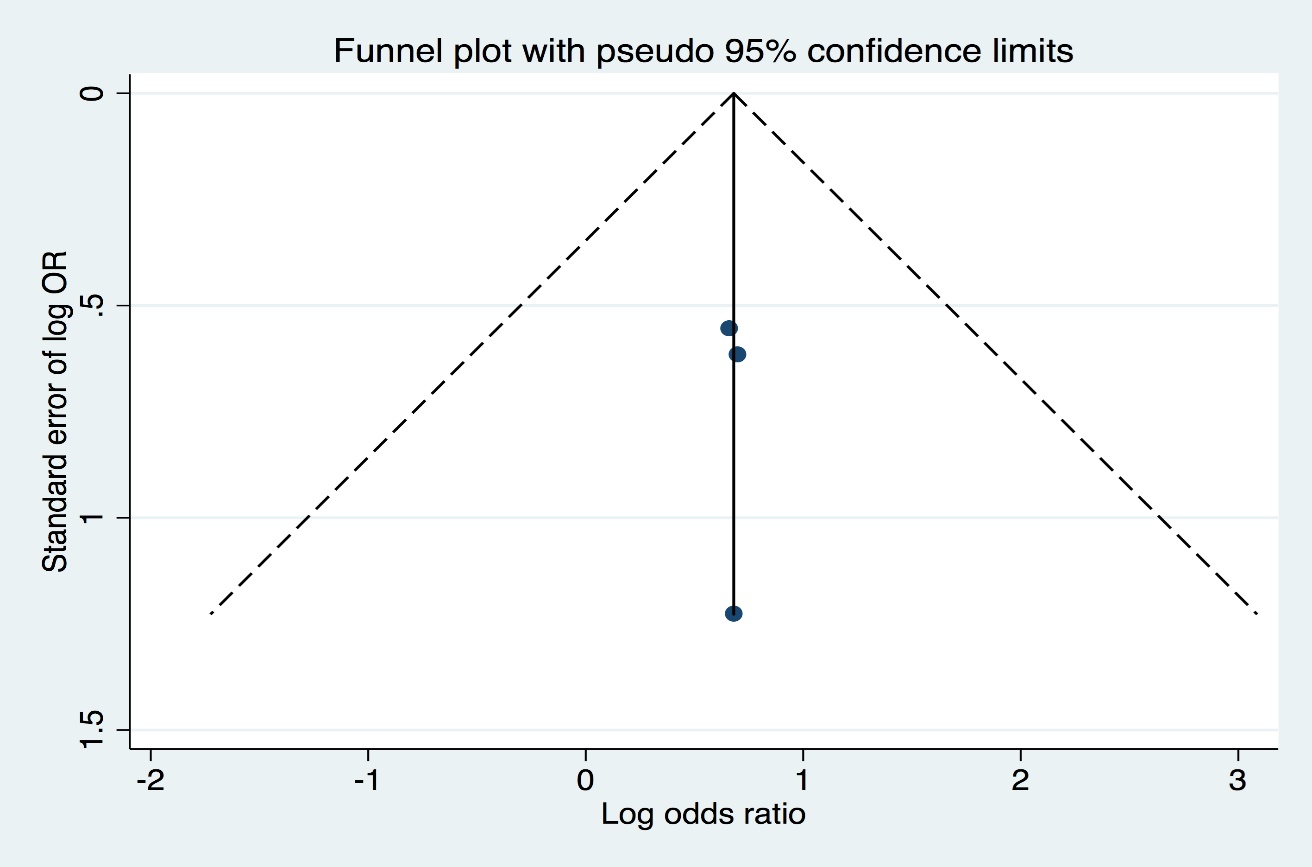


**Figure S4.** Funnel plot for the major bleeding events outcome in the thromboprophylaxis studies


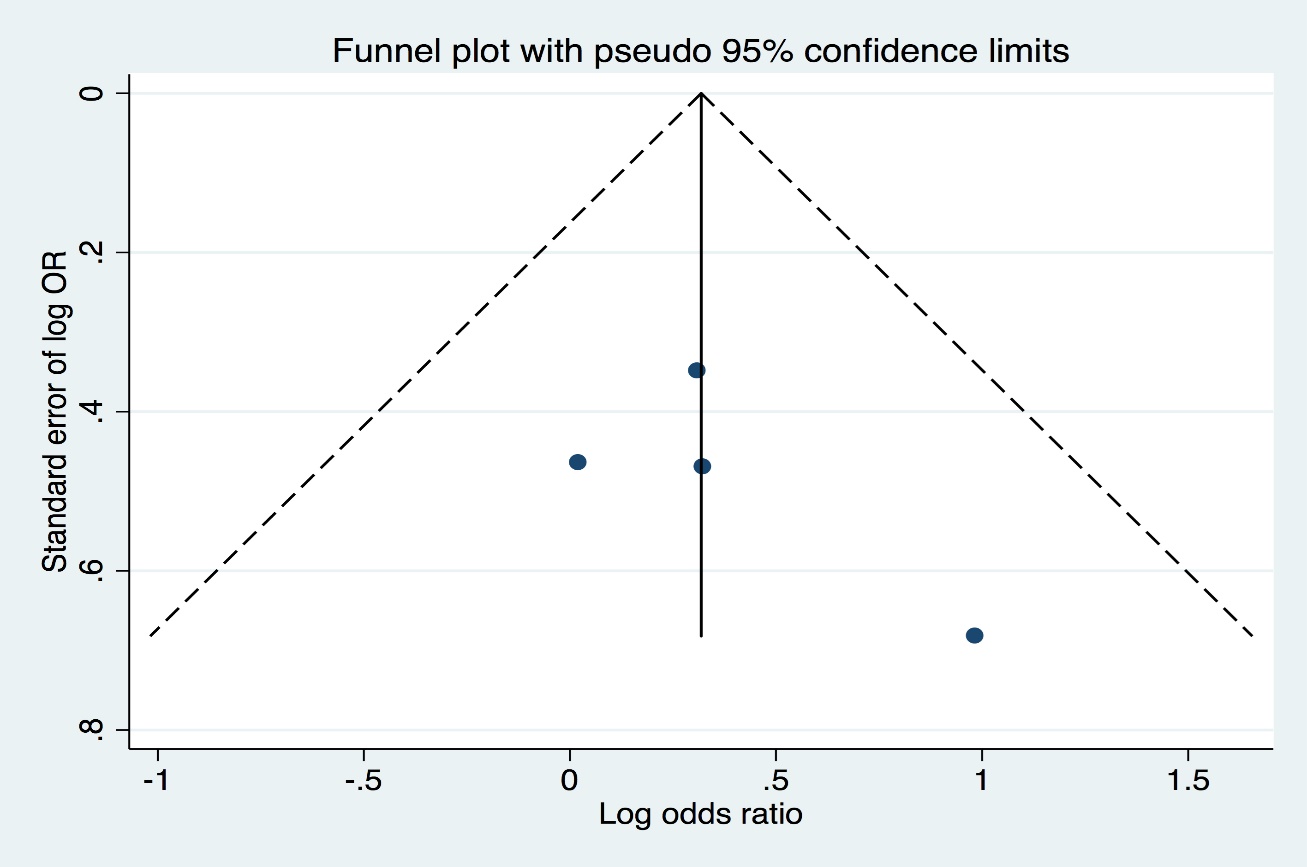


**Figure S5.** Funnel plot for the clinically relevant nonmajor bleeding events outcome in the thromboprophylaxis studies


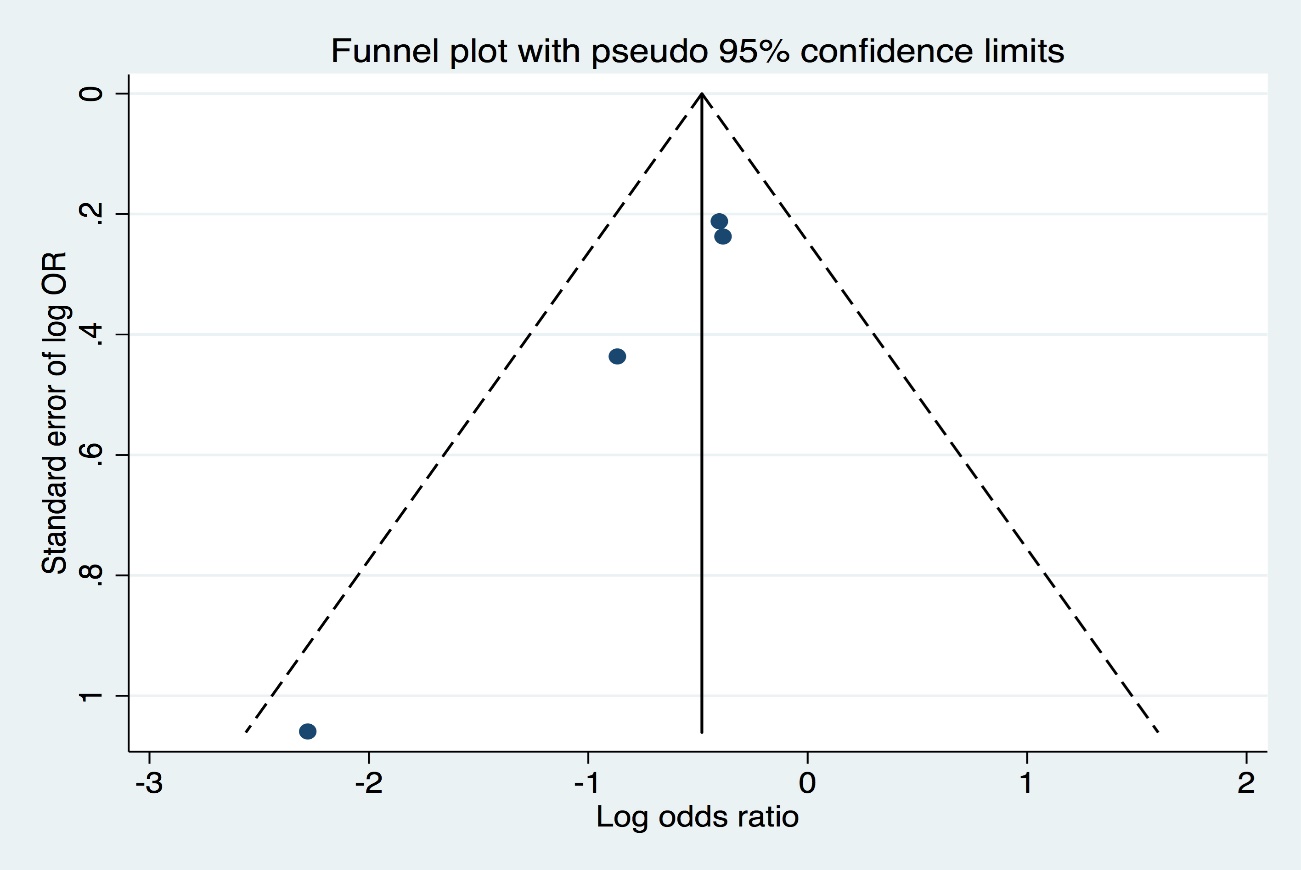


**Figure S7**. Funnel plot for the VTE recurrence outcome in the treatment studies


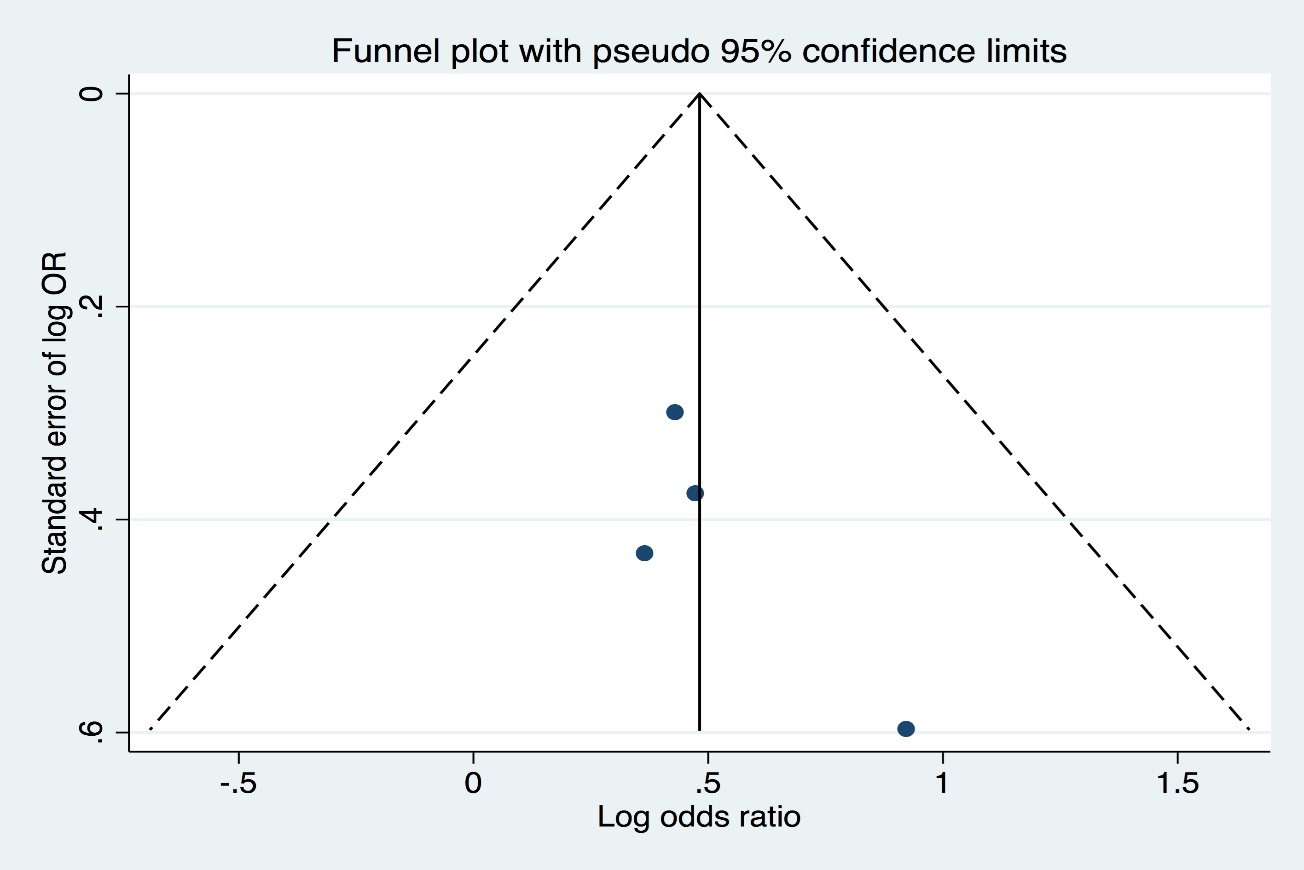


**Figure S6.** Funnel plot for the major or clinically relevant nonmajor bleeding events outcome in the thromboprophylaxis studies


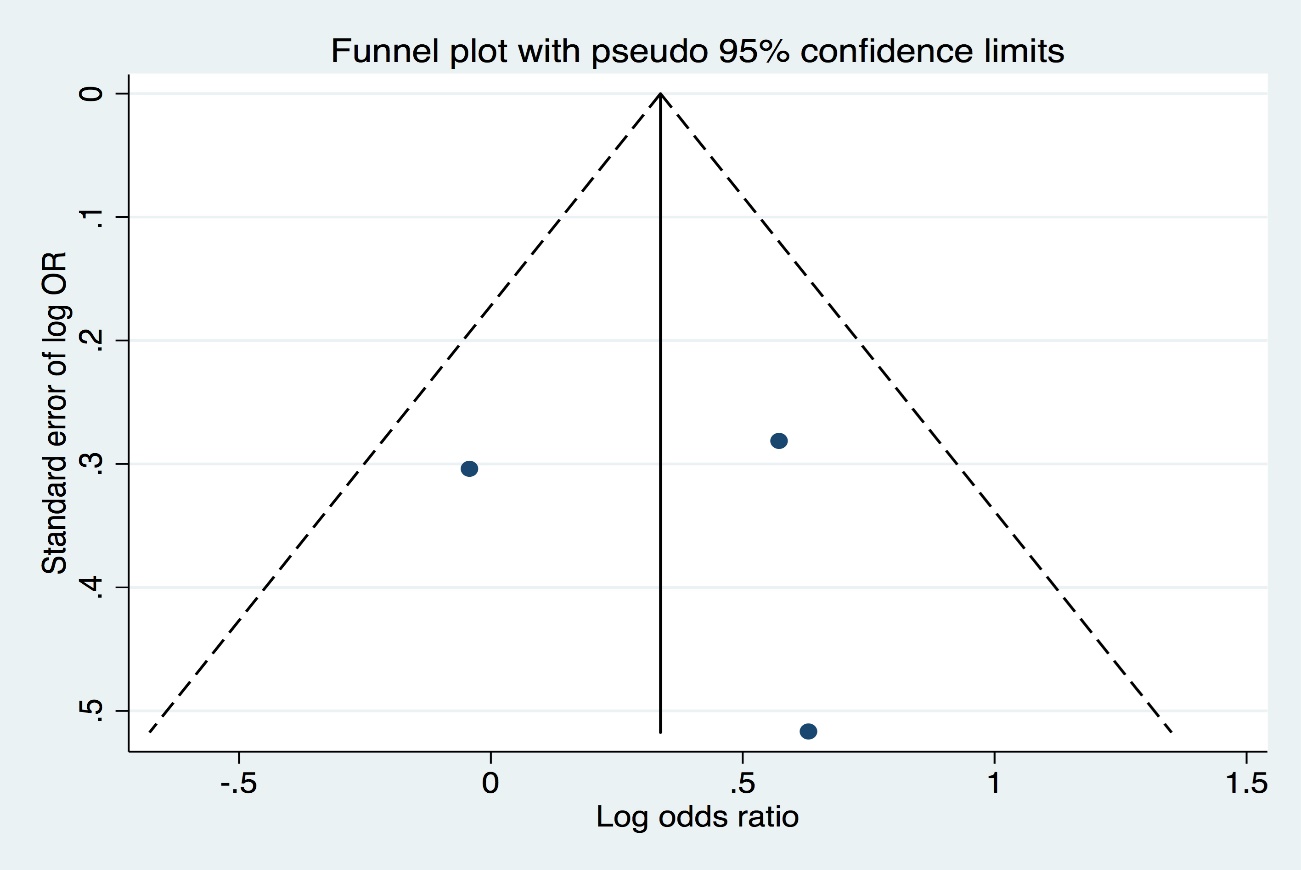


**Figure S8.** Funnel plot for the major bleeding events outcome in the treatment studies


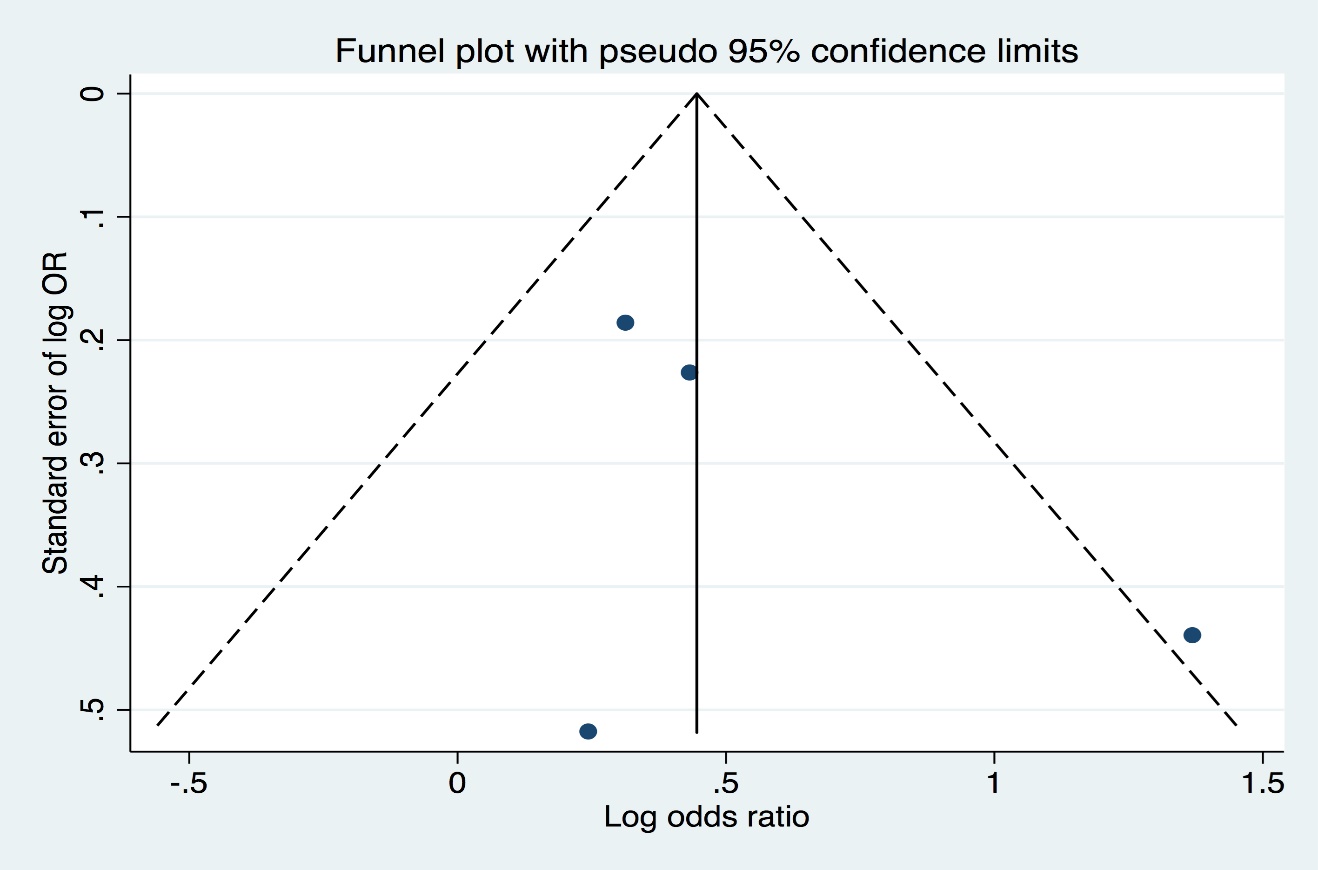


**Figure S9.** Funnel plot for the clinically relevant nonmajor bleeding events outcome in the treatment studies


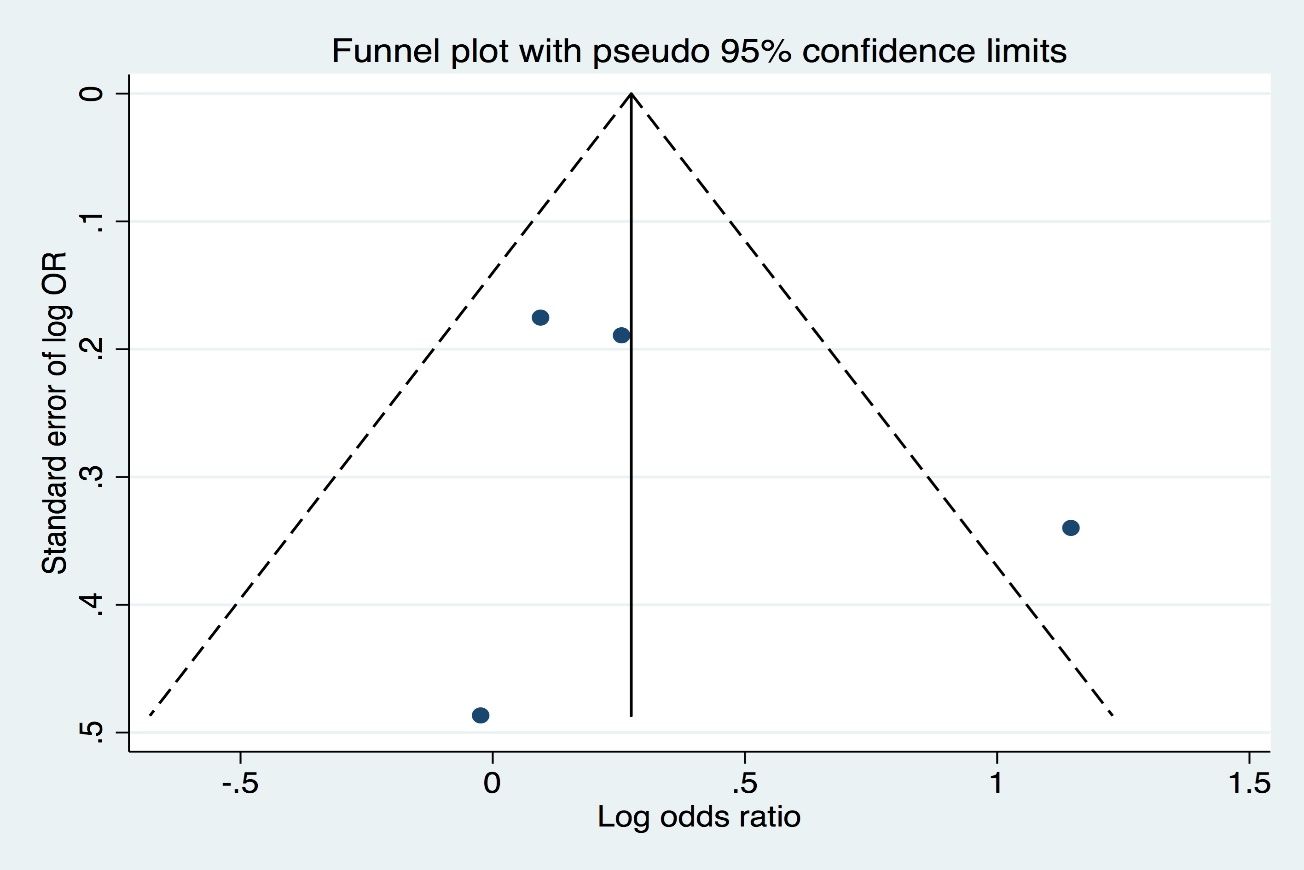


**Figure S10.** Funnel plot for the major or clinically relevant nonmajor bleeding events outcome in the treatment studies


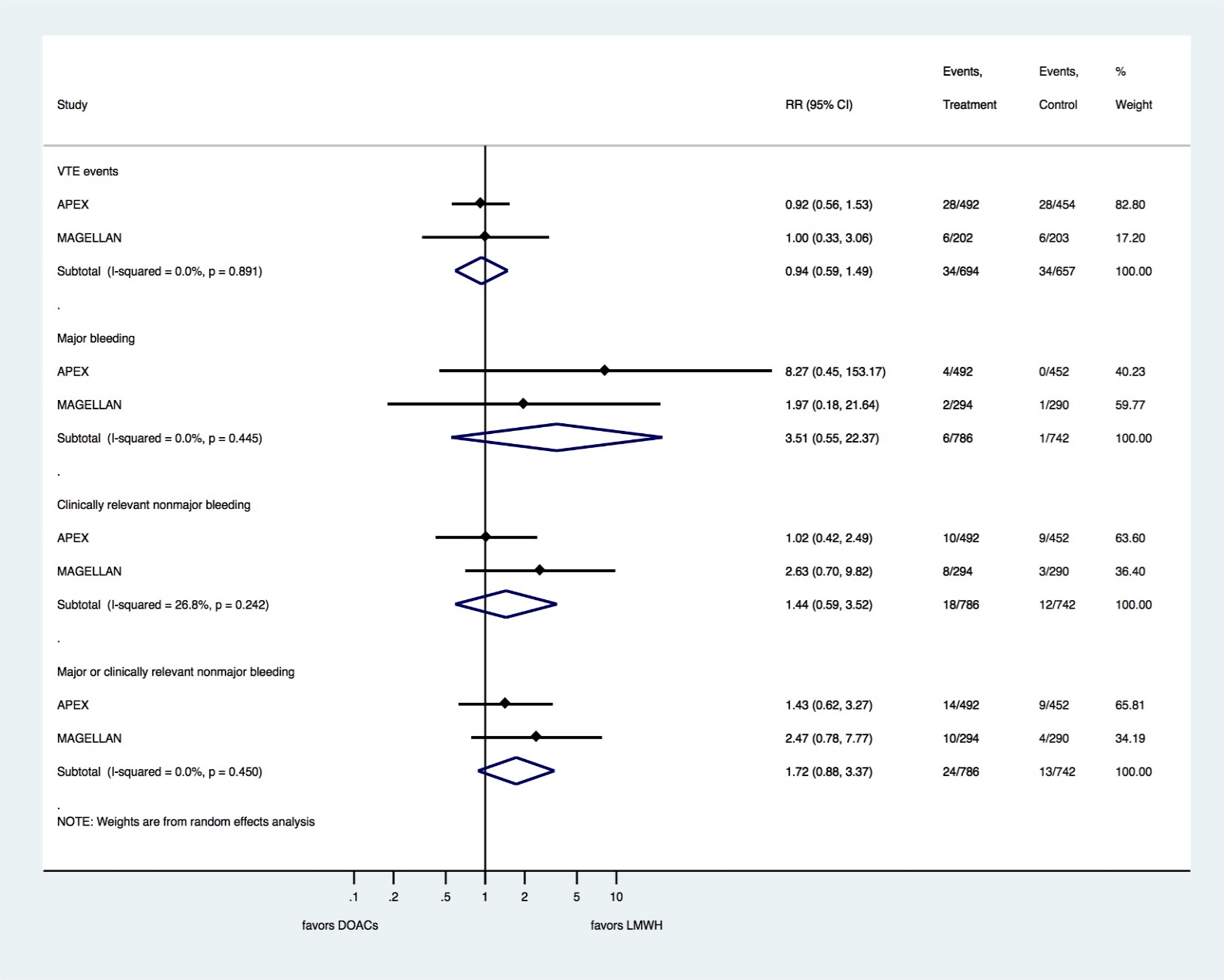


**Figure S11**. Thromboprophylaxis results (DOACs vs. LMWH)


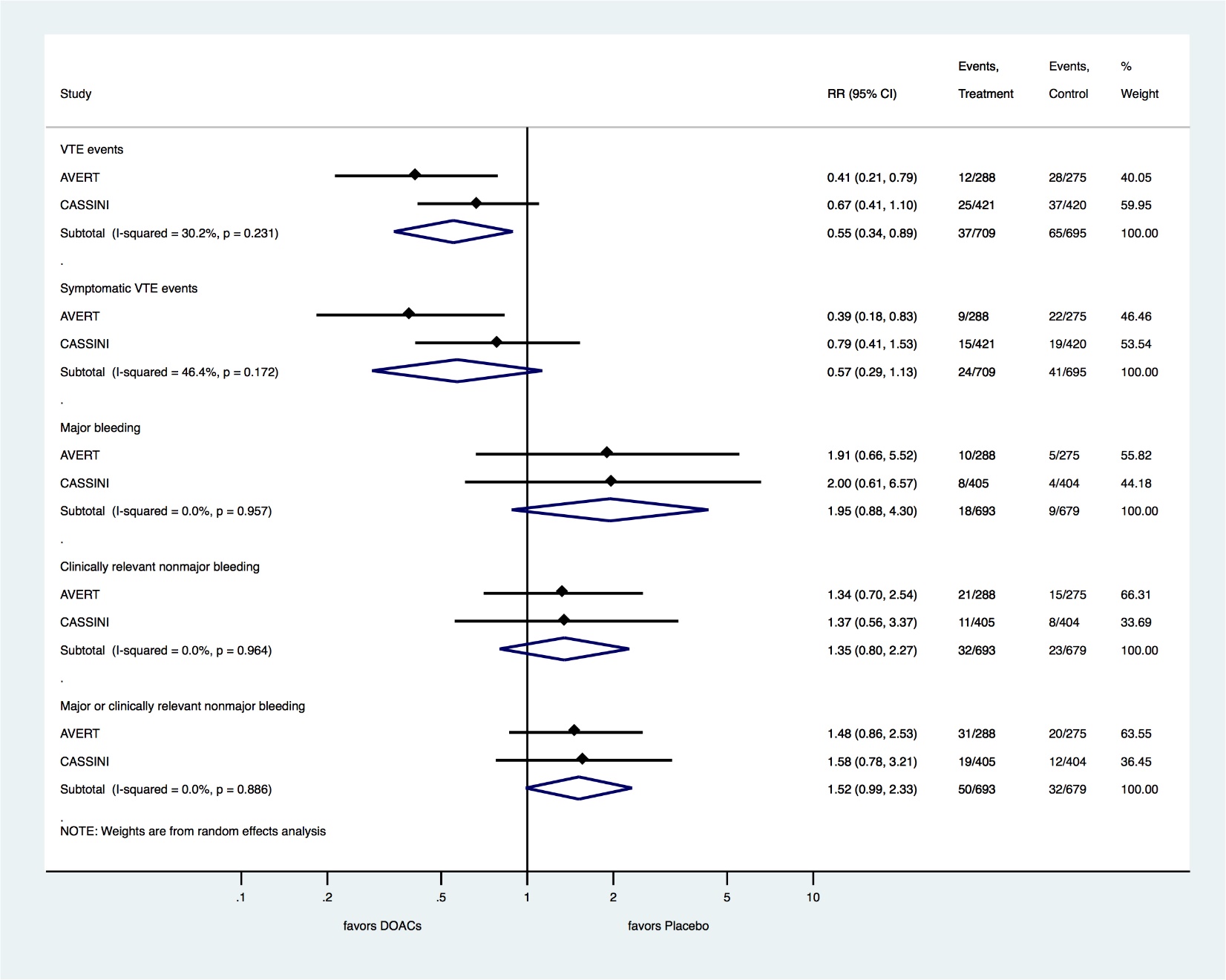


**Figure S12.** Thromboprophylaxis results (DOACs vs. placebo)
